# Relationship between red blood cell aggregation and dextran molecular mass

**DOI:** 10.1038/s41598-022-24166-w

**Published:** 2022-11-17

**Authors:** Maciej Bosek, Blanka Ziomkowska, Jerzy Pyskir, Tomasz Wybranowski, Małgorzata Pyskir, Michał Cyrankiewicz, Marta Napiórkowska, Maciej Durmowicz, Stefan Kruszewski

**Affiliations:** 1 Biophysics Department, Collegium Medicum of Nicolaus Copernicus University, Jagiellońska St. 13, 85-067 Bydgoszcz, Poland; 2 Department of Rehabilitation, Collegium Medicum in Bydgoszcz, Nicolaus Copernicus University, Toruń, Poland; 3 Department of Physiotherapy, Collegium Medicum in Bydgoszcz, Nicolaus Copernicus University, Toruń, Poland

**Keywords:** Biophysics, Haematological diseases

## Abstract

The aim of this study was to investigate the aggregation of red blood cells (RBCs) suspended in dextran solution at various levels of molecular mass. Dextran solutions at molecular mass 40, 70, 100 and 500 kDa at concentration from 2 to 5 g/dL were used to suspend the RBCs. The radius and velocity of sedimenting RBC aggregates were investigated using image analysis. The radius and sedimentation velocity of aggregates increased initially, then decreased after achieving maxima. The maximal velocity of RBC aggregates showed a bell-shaped dependence on dextran molecular mass and concentration, whereas maximal radius showed monotonic increase with both factors. Difference between aggregate and solution density was estimated using aggregate radius and sedimentation velocity and dextran solution viscosity, and was consistent across most molecular mass and concentration levels. This allowed to calculate the porosity of aggregates and to show that it monotonically decreased with the increase in the solution density, caused by the increase in the dextran concentration. The results provide insight into the RBC aggregation process in solutions of proteins of different size, reflecting various pathological conditions. The currently reported data can be potentially applied to specific pathophysiological conditions giving an interpretation that is not yet fully discussed in the literature.

## Introduction

The aggregation of red blood cells (RBCs) is a result of interaction between the cells and surrounding fluid and provides important diagnostic information about human blood. RBCs aggregation is of great medical importance in haemostasis and thrombosis^1,2^. In many pathological conditions, an increase in high-molecular-weight protein e.g., fibrinogen (340 kDa) and immunoglobulin G (150 kDa), and advanced oxidation protein products (AOPPs) are observed. In addition, recent studies have also shown an increase in fibrinogen concentration and AOPPs levels in patients infected with the SARS-CoV-2 virus^3,4^. It seems important to find the dependence between the amount of larger proteins such as fibrinogen and immunoglobulin G, or protein aggregation in the blood and higher RBCs aggregation. Indirectly, this may explain the higher incidence of thrombosis and blood rheological disturbances in these patients^3^.

The aggregation begins with rouleau formation. Many experimental and theoretical studies starting from Ponder^5^ have been dedicated to this initial phase of aggregation^6–9^. In the next phase, branched rouleaux appear which finally form 3D RBC aggregates. At higher hematocrit levels a rouleaux network appears. 3D RBC aggregates due to gravity sediment creating a base deposit at the bottom. Finally, only single erythrocytes and small aggregates remained above the deposit. First complex study of the 3D RBC aggregate formation and their sedimentation was given by Kernick et al.^10^. These stages of the aggregation process later have been studied extensively using light scattering^6,11,12^ and transmission^13,14^, ultrasound backscattering^15–17^, electrical measurements^18^, atomic force microscopy^19^, cell surface affinity measurements^20^, microscopic observations^7,13,21–23^ and settling^24–28^. Theoretical and numerical investigations on aggregation mechanisms have been also conducted^8,29–33^. Knowledge about RBC aggregation has been significantly increased, but some fundamental aspects remain unclear.

A natural feature of 3D aggregates is porosity, which is a widely observed phenomenon in many fields^34–36^. It affects the rheological properties of structures by decreasing their effective density, and by allowing solution to flow through these structures^37^. Investigation of the aggregate porosity as a function of size has been revealed the fractal geometry of the aggregates estimating their fractal dimension^13,22,23,27,28,37^.

There are two models of RBC aggregation^38^. First, the bridging model assumes that two adjacent RBCs are joined via fibrinogen or polymer bridges attached to the RBC glycocalyx. Second, the depletion model assumes that the concentration of fibrinogen or polymer is reduced in plasma or polymer solution near the RBC membrane. Resulting osmotic pressure triggers co-attraction of adjacent RBCs. Numerous studies of the aggregation process in dextran solutions have found it to be dependent on concentration and molecular mass of dextran^14,19–21,39–41^, both dependencies showing bell-shaped functions. The peak of aggregation appears at dextran concentration of approximately 3–4 g/dL and molecular mass 500 kDa^14,19,20^. In the depletion model the initial increase of interaction energy between RBCs with increasing concentration is due to increased osmotic pressure difference, whereas further decrease is caused by reduced depletion layer thickness^32^. In this model the changes of interaction energy between RBCs with increasing dextran molecular mass are also due to changes of these factors^14,31^. The bridging model offers various explanations for decreased aggregation after the peak with increasing dextran concentration, including electrostatic repulsion between RBCs^20^ or a change in dextran conformation with its concentration^21^.

The aim of this study was to investigate the effect of dextran concentration and molecular mass on the properties of arising RBC aggregates. Their velocity and sizes were obtained using an innovative method based on the analysis of consecutive images of settling objects. Next, based on these parameters, the structure of RBC aggregates was determined.

## Materials and methods

The venous blood used in this study was drawn in the local blood center from healthy adult volunteers. The blood was collected on K_3_EDTA anticoagulant. The study was conducted according to the guidelines of the Declaration of Helsinki, and approved by the Ethics Committee Nicolaus Copernicus University in Toruń, Collegium Medicum in Bydgoszcz (KB 116/2011). Written informed consent was obtained from the patients.

The RBCs were extracted by centrifugation of the whole blood at 3000 rpm for 5 min at 4 °C then removing the plasma and buffy coat. The RBCs were washed three times, centrifuging them in solution of phosphate buffered saline (PBS). Suspensions of RBCs in dextran solutions at hematocrit 5% and 10% were investigated. Each suspension contained RBCs from one donor. The solutions of dextran 40, 70, 100 and 500 kDa in PBS at concentrations of 2, 3, 4 and 5 g/dL were used. For each combination of hematocrit and dextran molecular mass and concentration at least five samples were measured. The sample from each individual donor was used only once in each combination. The sample was shaken for 5 min and injected into a rectangular glass-walled container of 18 mm width × 23 mm height × 1 mm depth. A charge-coupled device camera captured the images at the center of the sample, uniformly back-illuminated using a white light-emitting diode lamp. The light illuminating the sample was scattered by the ground glass. Images of an area 2.9mm^2^ (1000 × 1000 pixels) were captured. One image was saved per second over a 50-min period. The temperature during the measurements was about 22 °C. The viscosity of dextran solution at each molecular mass and concentration level was measured using the rotational viscometer (DV-1P; Anton Paar).

**Figure 1:**
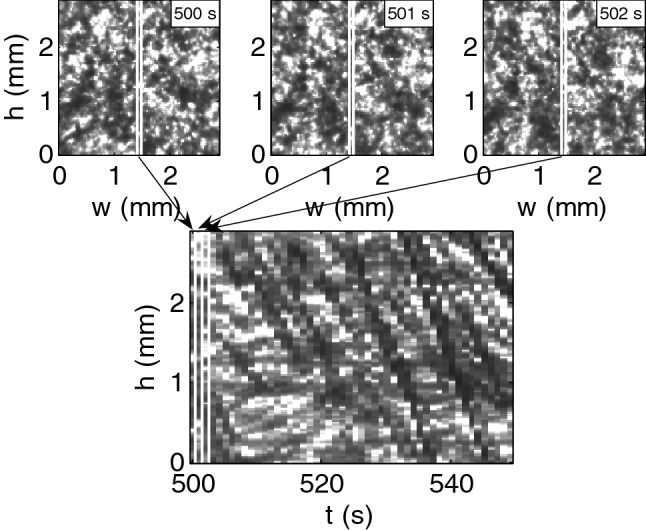
The procedure by which the image time sequence (bottom) was composed from the consecutive images of red blood cell (RBC) suspension (top) at hematocrit 10% in solution of dextran 70 kDa at concentration 3 g/dL.

The velocity and size of 3D aggregates were measured as a function of time. Both were obtained by analysis of a time series of images using a method described in our earlier study^28^. To determine the velocity, the clippings of one pixel width of 50 consecutive images covering the same column of the imaged sample area were composed in time sequence. This procedure is illustrated in Fig. 1. Each line on the time sequence (Fig. 1, bottom plot) represents the vertical movement of aggregate within given column, and the slope of the line determines the aggregate velocity.

**Figure 2:**
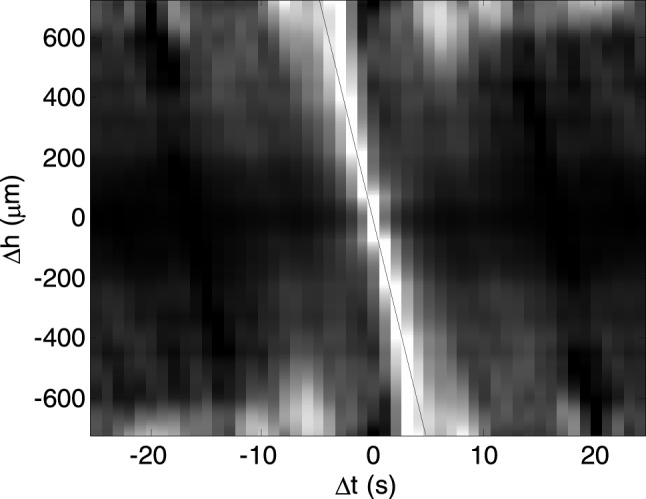
The autocorrelation of time sequence as a function of sample height shift and time shift, averaged over 50 s time interval and whole imaged area of the sample of the red blood cell (RBC) suspension at hematocrit 10% in solution of dextran 70 kDa at concentration 3 g/dL. The slope of the linear fit is an estimation of the mean aggregate velocity.

To estimate mean slope, an autocorrelation of these time sequence as a function of sample height shift and time shift, averaged over all columns, was calculated. Figure 2 shows an example of this function. The slope of the line along the peak of this function was obtained, using the least squares method in Fourier space^26–28^, yielding an estimate of the mean aggregate velocity.

**Figure 3:**
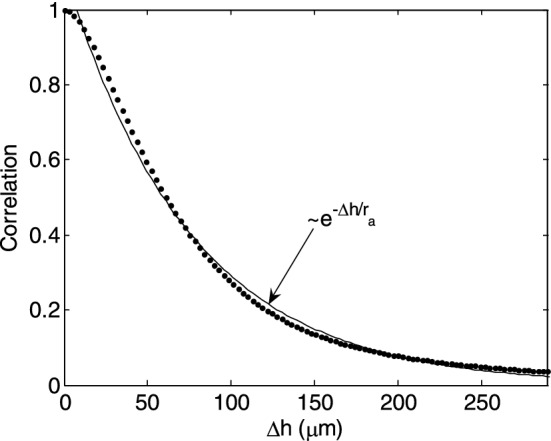
The correlation of the images as a function of sample height shift, averaged over 50 s time interval and whole imaged area of the sample of the red blood cell (RBC) suspension at hematocrit 10% in solution of dextran 70 kDa at concentration 3 g/dL, which is the cross section of the autocorrelation function shown in Fig. 2 at Δ*t* = 0. The parameter *r*_*a*_ of the exponential fit (solid line), was taken as a measure of the mean aggregate radius.

Using the autocorrelation function described above, the aggregate size may also be determined. The cross section of this function at a time shift of zero is the autocorrelation of the images as a function of sample height shift. Figure 3 shows an example of the autocorrelation as a function of height only. This function describes the size of the areas covered by aggregates and gaps between them.

However, we assume that the size of the gaps is substantially greater than that of the aggregates, so for small distances only the latter are represented. Since this function exhibits an exponential decay, the distance at which exponential fit to this function decreased *e*-times was taken as a measure of the mean aggregate radius. Both parameters are averaged over the 50 s consecutive time interval and the whole imaged area.

**Figure 4:**
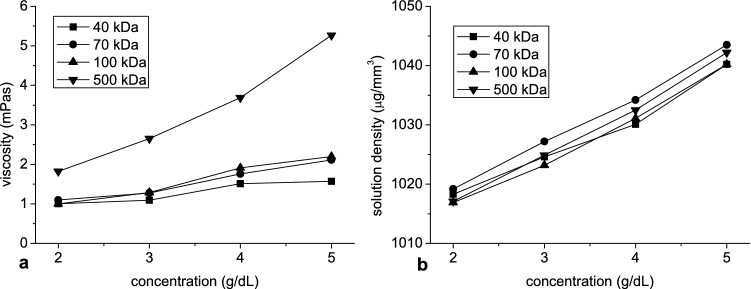
The mean of the solution viscosity (**a**) and density (**b**) as a function of dextran concentration.

To describe of the structure of sedimenting aggregate using obtained parameters their terminal velocity was considered. At high Peclet number and low Reynolds number, it is given by:

$$ v_{S} = \frac{{2gr_{a}^{2} {\Delta }\rho }}{9\mu } $$

where g is the acceleration of gravity, Δρ is a difference between aggregate and solution density, μ is viscosity of dextran solutions shown in Fig. 4a and *r*_*a*_ is aggregate radius. It should be noted that erythrocytes or rouleaux whose shape is far from spherical are too small to be detected by the method used herein and only larger aggregates which are approximately spherical are tracked. The above terminal velocity was calculated for an isolated aggregate, whereas in this study velocity of many hydrodynamically interacting aggregates, was measured. These sedimenting aggregates generated fluid backflow, in turn decreasing their velocity. This hindrance effect, according to Richardson and Zaki, is given by:^42^

$$ v_{S} = \frac{{v_{a} }}{{\left( {1 - H} \right)^{4.65} }} $$

where *H* is the hematocrit and *v*_*a*_ the velocity of aggregates. If density difference for peak radius is considered, as *v*_*a*_ the peak velocity is accepted, since times to peak both of them were very close and it was assumed that largest aggregates reached highest velocity. Finally, the difference between aggregate and solution density is given by:

$$ {\Delta }\rho = \frac{{9\mu v_{a} }}{{2gr_{a}^{2} \left( {1 - H} \right)^{4.65} }}. $$

Finally the aggregate porosity can be obtained. It is the ratio of volume occupied by solution between erythrocytes in the RBC aggregate to the overall aggregate volume and is given by:^37^

$$ p = 1 - \frac{{{\Delta }\rho }}{{\rho_{e} - \rho_{s} }} $$

where *ρ*_*e*_ is the averaged erythrocyte density equal to 1110 µg/mm^3^ and *ρ*_*s*_ is solution density shown in Fig. 4b. The erythrocyte density was assumed to be independent of the suspending solution.

## Results

**Figure 5:**
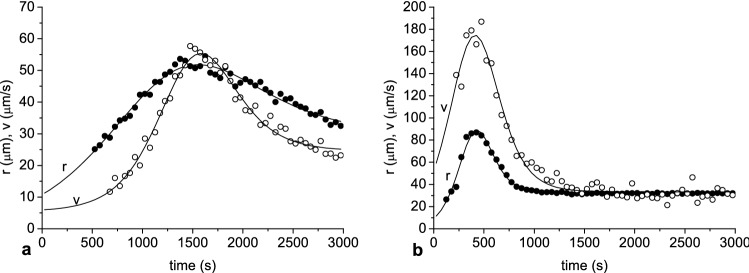
The aggregate radius and velocity as a function of time at hematocrit 5% in solution of dextran 40 kDa at concentration 5 g/dL (**a**) and at hematocrit 10% in solution of dextran 70 kDa at concentration 3 g/dL (**b**). The solid lines represent an experimental function fitted to these data.

Figure 5 shows the aggregate radius and velocity as a function of time. After initial variation, these parameters showed low values, then peaked and decreased. The dynamics of changes in radius as well as velocity of RBC aggregates varied with dextran molecular mass and concentration. To characterize this dynamic, the experimental function was fitted to these dependencies. The best fit was provided by a combination of bell-shaped and sigmoid functions. The curve fitting procedure allowed estimation of the maximal (peak) values of aggregate radius and velocity, and times at which these values were achieved.

**Figure 6:**
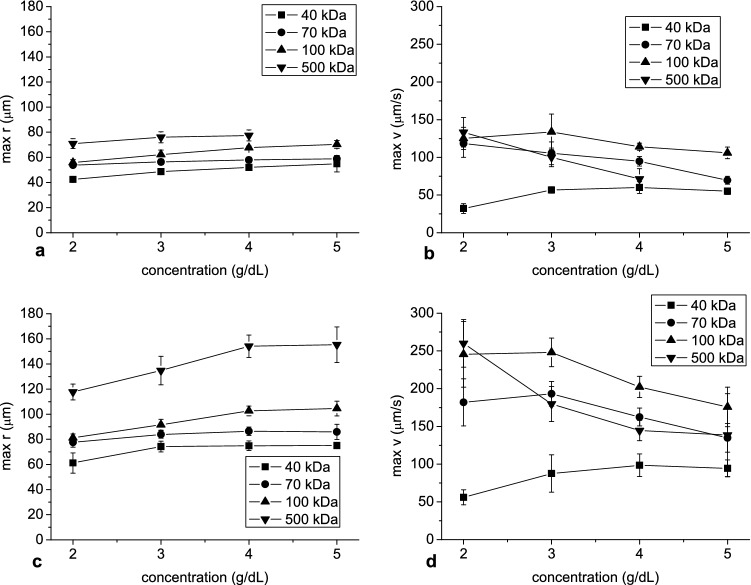
The mean and standard deviation of the peak aggregate radius (**a**, **c**) and velocity (**b**, **d**) as a function of dextran concentration at hematocrit 5% (**a**, **b**) and 10% (**c**, **d**). In this and next figures the standard deviations were calculated for the values over different experiments.

**Figure 7:**
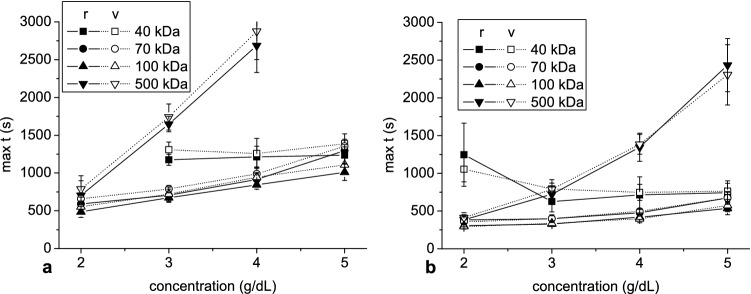
The mean and standard deviation of the time to reach peak aggregate radius and velocity as a function of dextran concentration at hematocrit 5% (**a**) and 10% (**b**).

The molecular mass and concentration of dextran affected the time evolution of mean aggregate radius and sedimentation velocity in the RBC suspensions. The dependence on dextran concentration is shown in Figs. 6 and 7. Peak aggregate radius increased with dextran concentration (Fig. 6a, c). In general, peak aggregate velocity decreased with increasing concentration, however, at low dextran concentration and molecular mass a peak increase was observed (Fig. 6b, d). An increase in time to peak aggregate radius as well as sedimentation velocity with increasing dextran concentration was found (Fig. 7).

**Figure 8:**
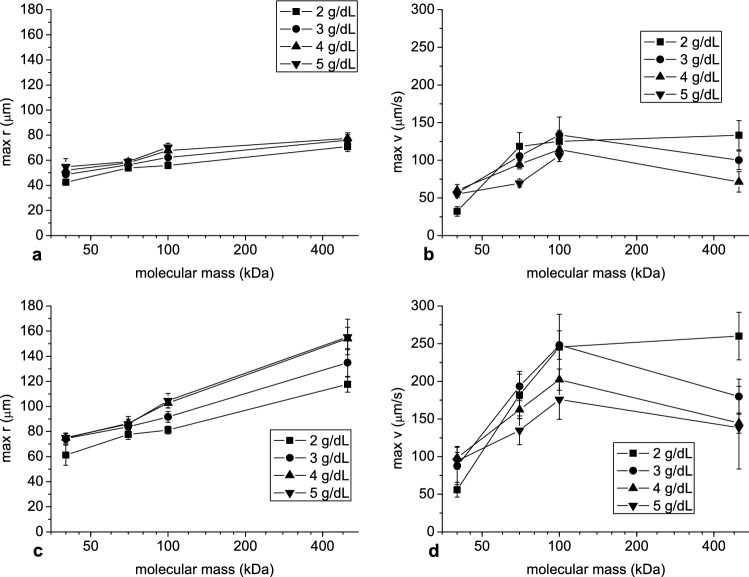
The mean and standard deviation of the peak aggregate radius (**a**, **c**) and velocity (b,d) as a function of dextran molecular mass at hematocrit 5% (**a**, **b**) and 10% (**c**, **d**).

**Figure 9:**
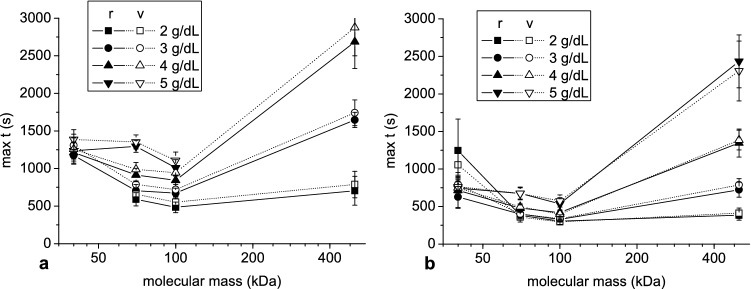
The mean and standard deviation of the time to reach peak aggregate radius and velocity as a function of dextran molecular mass at hematocrit 5% (**a**) and 10% (**b**).

The dependence of outcome parameters on dextran molecular mass, shown in Figs. 8 and 9, was more complex. Peak aggregate radius showed constant growth with the logarithm of dextran molecular mass (Fig. 8a, c) while peak velocity initially increased with dextran molecular mass and, with the exception of dextran concentration 2 g/dL, reached maximum at 100 kDa, then decreased (Fig. 8b, d). The time to peak radius and velocity initially decreased and, after reaching a minimum at 100 kDa, increased with molecular mass (Fig. 9).

**Figure 10:**
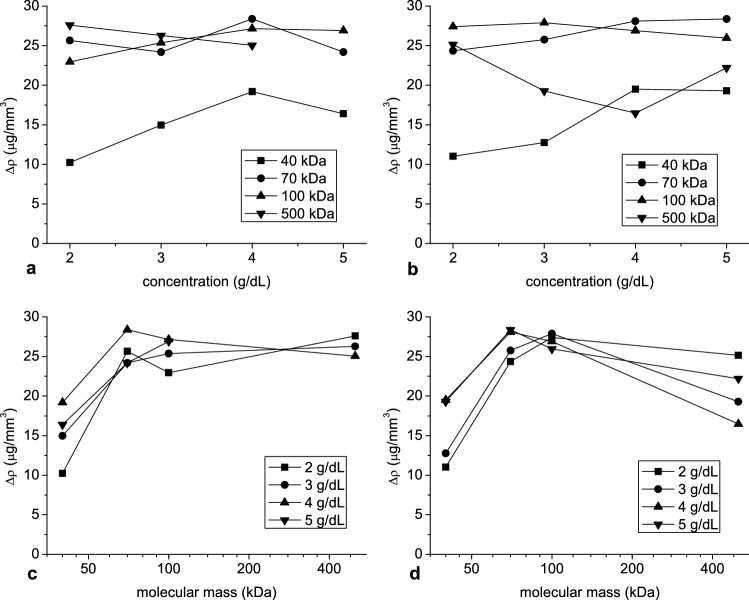
The mean of the difference between aggregate and solution density at hematocrit 5% (**a**, **c**) and 10% (**b**, **d**) as a function of dextran concentration (**a**, **b**) and molecular mass (**c**, **d**).

**Figure 11:**
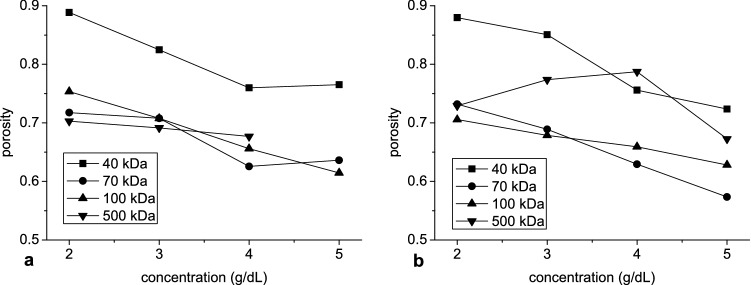
The mean of the aggregate porosity at hematocrit 5% (**a**) and 10% (**b**) as a function of dextran concentration.

In Fig. 10 the difference between aggregate and solution density is shown at aggregate peak radius versus dextran concentration and molecular mass. From 70 kDa molecular mass, this difference was close to 25 µg/mm3, almost independent of dextran molecular mass and concentration. Only at 500 kDa and hematocrit 10% it showed bells-shaped dependence from dextran concentration with a minimum at 4 g/dL. The density difference at 40 kDa was substantially smaller and increased with concentration. Next the porosity at aggregate peak radius was calculated and was shown versus dextran concentration in Fig. 11. The monotonic decrease of porosity with increasing dextran concentration was observed. This was found for all investigated molecular masses except 500 kDa at hematocrit 10%.

## Discussion

This study investigated the time dependence of size and sedimentation velocity of RBC aggregates, both of which exhibited two phases, initial increase then decrease. This behavior is a result of the coexistence of two processes, the first of which, aggregation, causes formation and increase in size of the aggregates. In the second process, sedimentation, growing aggregates settle. As the largest aggregates leave the observed region the fastest, sedimentation decreases the mean aggregate velocity and size. In the first phase, aggregation outweighs sedimentation, and in the second inversely.

Peak value and the time to reach this value were used to describe the time dependence of both aggregate radius and velocity. Apart from peak velocity, the relationship between these parameters and dextran concentration was monotonic, in contrast with other studies which have reported bell-shaped dependence of RBC aggregation on dextran concentration^19–21,39–41^. The monotonic increase of aggregate peak radius with dextran concentration observed in our study, along with monotonic increase in time to achieve peak radius and velocity, even above concentration at which maximum of aggregation was expected, may be explained by the longer time available for aggregation, which in turn was due to an increase in viscosity and density of suspending solution with dextran concentration. In general, peak velocity increased with concentration then decreased, the latter resulting in the above-mentioned longer duration for greater dextran concentration. The increase of aggregate velocity at lower dextran concentration was due to increased aggregate radius, whereas the decreased velocity at higher concentration was due to an increase in dextran solution viscosity and density. This behavior of aggregate velocity reflects the mechanisms responsible for bell-shaped dependence of aggregation on dextran concentration observed in other studies.

Three of the four parameters describing temporal evolution of mean aggregate radius and velocity exhibited typical bell-shaped dependence on dextran molecular mass. While the curve was bell-shaped for peak velocity, it was a similar but inverted curve for time to peak radius and to peak velocity indicating that the former implicated the latter. The initial increase and later decrease in aggregate velocity with increasing molecular mass may be explained by the increase in aggregate size and in dextran solution viscosity, respectively. This reflects the mechanisms underpinning the widely reported bell-shaped dependence of aggregation on dextran molecular mass, similar to those found for dependence on dextran concentration. Peak aggregate radius showed other dependence on dextran molecular mass. This parameter increased with molecular mass over the range included in this study. Similarly to its relationship with dextran concentration, it did not decreased for greater dextran molecular mass. This finding, along with increase in time to this peak, may be an effect of the prolonged process duration.

Difference between aggregate and solution density was calculated to aid interpretation of the relationship between peak aggregate radius and velocity and dextran solution viscosity. Density difference at this peak showed usually similar values across the range of concentrations and dextran molecular masses above 40 kDa. Behavior at 40 kDa may reflect the fact that at this molecular mass RBC aggregation is already low^31,38^. These results suggest that at peak radius and velocity when sedimentation prevails over aggregation, aggregate buoyancy is similar across molecular mass and concentration levels. Obtained density difference together with erythrocyte and solution density allow to calculate the aggregate porosity. In general, across the range of molecular masses the aggregate porosity at peak radius and velocity decreased with increasing dextran concentration. Since this peak was usually reached at similar density difference, an increase in the solution density with increasing concentration causes that the aggregates had to be increasingly packed in order for their sedimentation to prevail over further aggregation. An increase in RBC aggregate packing with an increase in the concentration of macromolecules was previously observed in numerical simulations^33^. Beyond the peak, however, dynamics yield non-monotonic changes in the fractal dimensions of RBC aggregates with increasing dextran concentration as we have reported previously^28^.

On the base of our data, it is reasonable to claim that high-molecular-weight proteins can influence the aggregation of RBCs. It was shown that the increase in dextran molecular mass causes the increase in the radius of aggregates, without considerable changes in their porosity. This suggests that with increasing dextran molecular mass within investigated range the RBC aggregation increased. This may also be true for conglomerates of oxidized albumin such as AOPPs. High-molecular-weight AOPPs are mainly aggregates of albumin created via disulfide bridges and/or dityrosine cross-linking^43,44^. The level of AOPPs is raised in many diseases, including renal failure, diabetes, atherosclerosis, breast and colon cancer, Alzheimer’s disease and chronic myeloid leukemia but also increases with age^45–50^.

Our experiment may also provide insight into the mechanism underlying thrombosis and the formation of blood clots in COVID-19 pathology and other acute infection. There is a strong link between the aggregation of RBCs and thrombosis^2,51,52^. Many studies have shown increased peripheral blood neutrophil activation with excessive development of Neutrophil Extracellular Traps (NETs) in COVID-19 patients due to aberrant immune response and higher virus replication^53–56^. It has been investigated that chlorine compounds produced by activated neutrophils in viral or bacterial infection lead to oxidative modification of albumin and the formation of AOPPs^57,58^. Indeed, the results of our previous study revealed that elevated level of AOPPs is associated with inflammation during the course of COVID-19-related pneumonia^59^. Interestingly, RBCs aggregation was also found to increase in patients with COVID-19^3^. In this study, patients with pulmonary lesions had higher aggregation of RBCs and enhanced coagulation.

We also hypothesize that the production of advanced lipoxidation end-products (ALEs-HSA) that formulate in reaction albumin with aldehyde may also contribute to the elevated aggregation of RBCs and thus participate in thrombosis events. Aldehyde products are created by lipid peroxidation due to oxidative stress in cells. It has been found that the modification of HSA with malondialdehyde (MDA) can also result in the aggregation of albumin^60^. Higher lipid peroxidation levels and increases in the concentration of aldehydes are associated with many diseases^61–63^. For example, hypoxic respiratory failure can contribute to the aggregation of albumin as hypoxic cells produce larger amounts of aldehydes and free radicals^64–66^. Also, the increased severity of the disease in COVID-19 patients was associated with lipid peroxidation levels^67,68^. In this light, the in-vitro tests of the influence of AOPPs or ALEs on RBCs aggregation seem to be urgently needed.

## Conclusion

This study increases understanding of the relationship between size and concentration of protein and the properties of arising RBC aggregates. It also enhances knowledge about dextran as a plasma substitute in medical treatment. The RBC aggregation at different dextran concentrations and different molecular weights may reflect the in-vivo behavior of erythrocytes in various pathological conditions, since in many diseases, there is an increase in the concentration of large proteins such as fibrinogen and the formation of protein aggregates. The differences of the structure of aggregates formed under different conditions, found here, therefore seems to be an important issue and should be further investigated.

## Data Availability

The data sets generated during and analyzed during the current study are available from the corresponding author on reasonable request.

## Abbreviations

RBCs: Red blood cells
PBS: Phosphate buffered saline
